# Supplementary material for: Loss of COX4I1 Leads to Combined Respiratory Chain Deficiency and Impaired Mitochondrial Protein Synthesis
Source: Cells. 2021 Feb 10;10(2):369. doi: 10.3390/cells10020369 (PMC7916595; doi:10.3390/cells10020369)
Supplement: Supplementary file 1 [file cells-10-00369-s001.pdf]

Supplementary material

Supplementary figure 1. Densitometric quantification of WB experiments a) Quantification of cIV subunits content normalized to actin. Data represent mean ± S.D. of wt (n=2), COX4I1 KO (n=4), COX4I2 KO (n=4), and COX4I1/4I2 KO cells (n=3). Statistically significant differences between wt and KO cells was calculated using ANOVA in GraphPad Prism 8. Asterisks (*) represent p-value: * < 0,05; ** < 0,01; *** < 0,001. **b)** Quantification of OXPHOS complexes subunits content normalized to actin. Data represent mean ± S.D. of wt (n=2), COX4I1 KO (n=4), COX4I2 KO (n=4), and COX4I1/4I2 KO cells (n=3). Statistically significant differences between wt and KO cells was calculated using ANOVA in GraphPad Prism 8. Asterisks (*) represent p-value: * < 0,05; ** < 0,01; *** < 0,001.

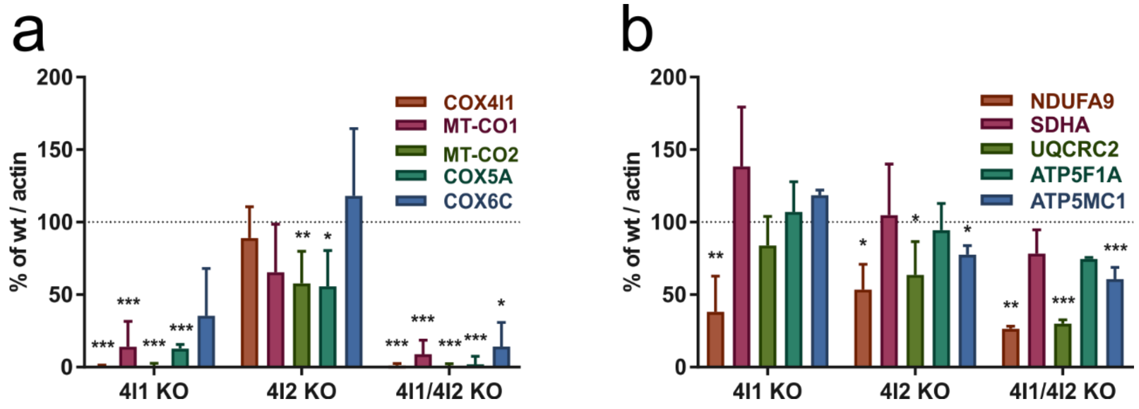

Supplementary figure 2. MS-LFQ proteomic analysis. **a)** Volcano plot depicting differential content of all detected proteins between wt and COX4i1/4i2 KO HEK293 cells. Relative quantities plotted on X-axis are expressed in log2 scale. Different colours depict proteins of individual OXPHOS complex, mitochondrial ribosomal proteins, other mitochondrial proteins (MitoCarta2.0-annotated), and the remainder of the cellular proteome. **b)** Average fold changes (log2 scale) of mitochondrial proteins. Data were calculated for all detected mitochondrial proteins (Mitocarta 2.0 annotated), group of OXPHOS proteins (including OXPHOS complexes subunits and their assembly factors), and proteins subunits of mitochondrial ribosomes (MRPs). **c)** Average fold changes (log2 scale) for subunits of OXPHOS complexes. Data were calculated from quantities of detected subunits of cI (n=38), cII (n=4), cIII (n=8), cIV (n=9), and cV (n=12).

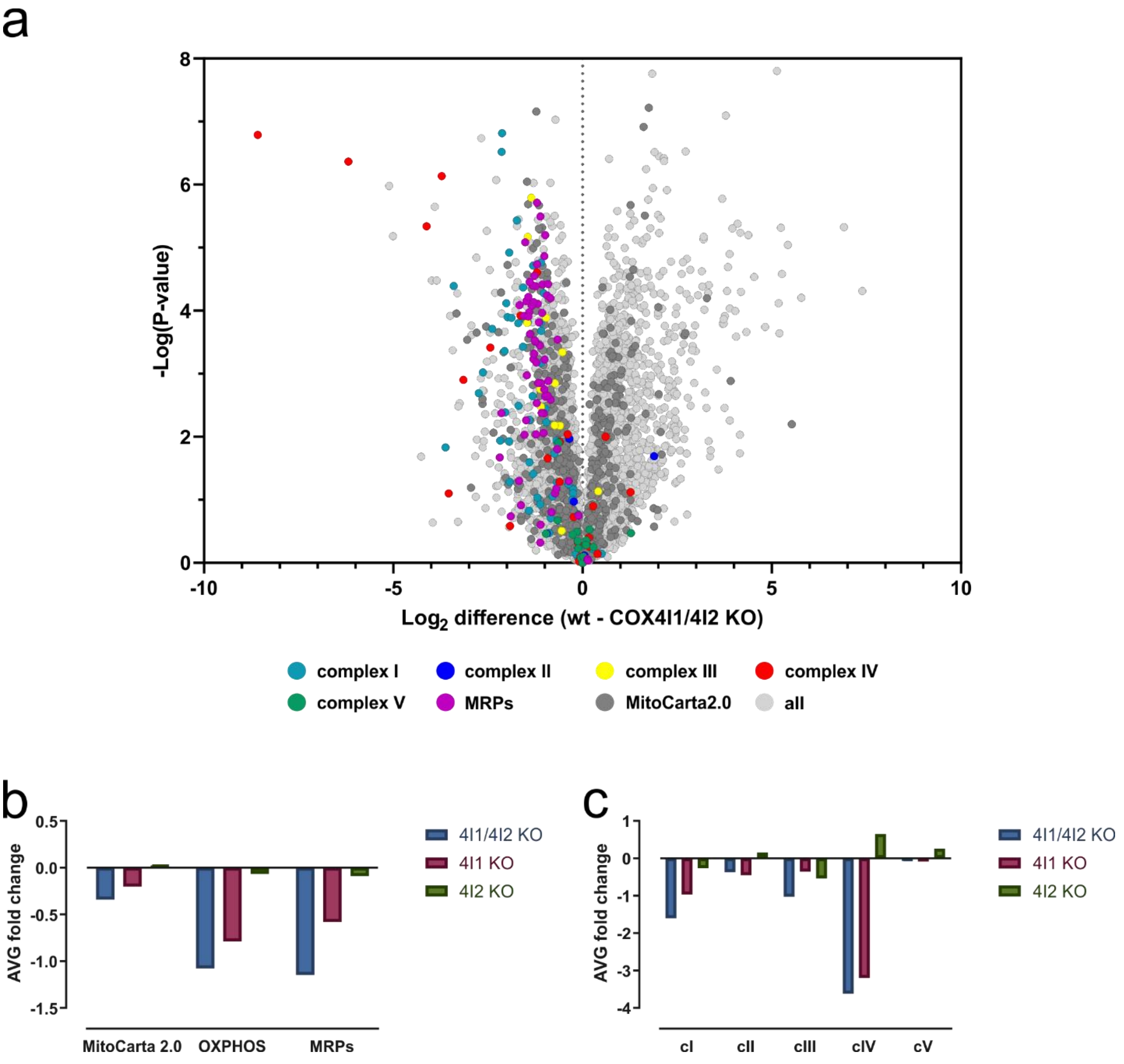

Supplementary figure 3. Complementation of COX4I1/4I2 KO by COX4I2 isoform. Digitonin solubilized mitochondria (30 µg of protein) were subjected to BN-PAGE and Western blot detection in parallel with antibody against MT-CO1 and antibody specific to COX4I2. COX4I2 signal was detected only in COX4I2 KI cells, similar band pattern to MT-CO1 detection indicates rescue of cIV assembly and supercomplex incorporation by the “hypoxic” isoform 2 of COX4.

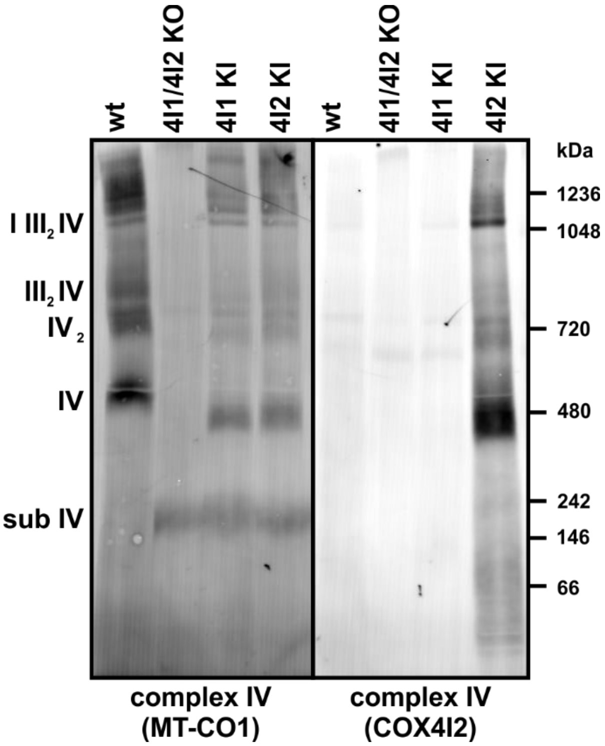

Supplementary figure 4. Complexome profiling analysis of cV content and assembly status. Heatmap displays relative content and migration of cV components under native conditions in control HEK293 and COX4I1/4I2 KO cells. Migration of the most prominent cV forms – dimer (V_D), monomer (V_M), and F_1 subassemblies (F_1) are indicated above the heatmap. Data represent relative peptide intensity profile of each protein normalized to the slice with the highest intensity, averaged from duplicate experiments, according to the colour scale below the heatmap.

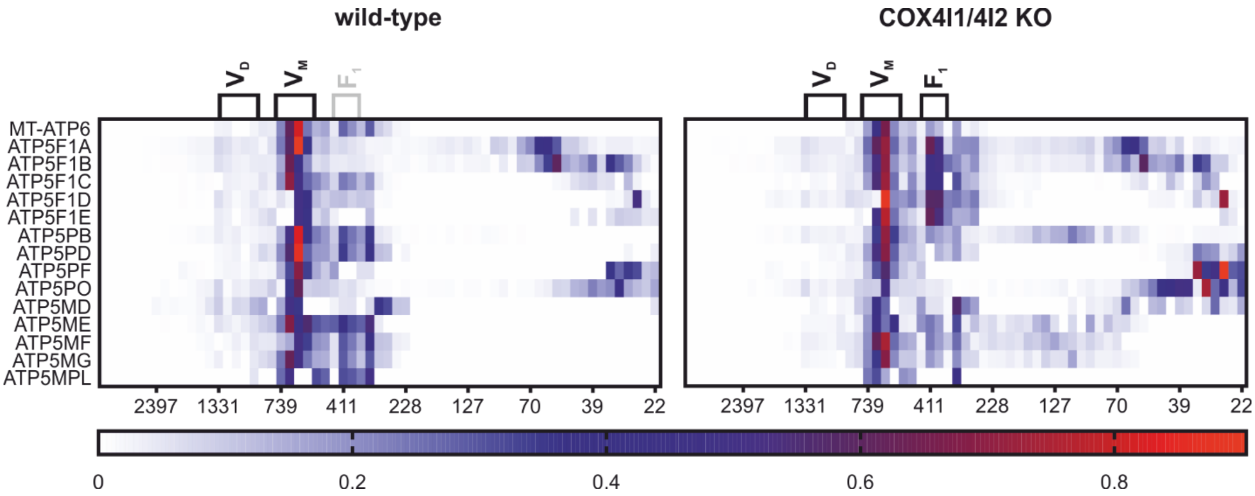

Supplementary figure 5. Quantitative analysis of pulse-chase experiments. Densitometric quantification of labelled MT-CO1, MT-CO2/MT-CO3, MT-CYB, and MT-ATP6 from autoradiographic images of pulse and 24-hour chase samples are represented as average values of two independent experiments. Error bars represent SD.

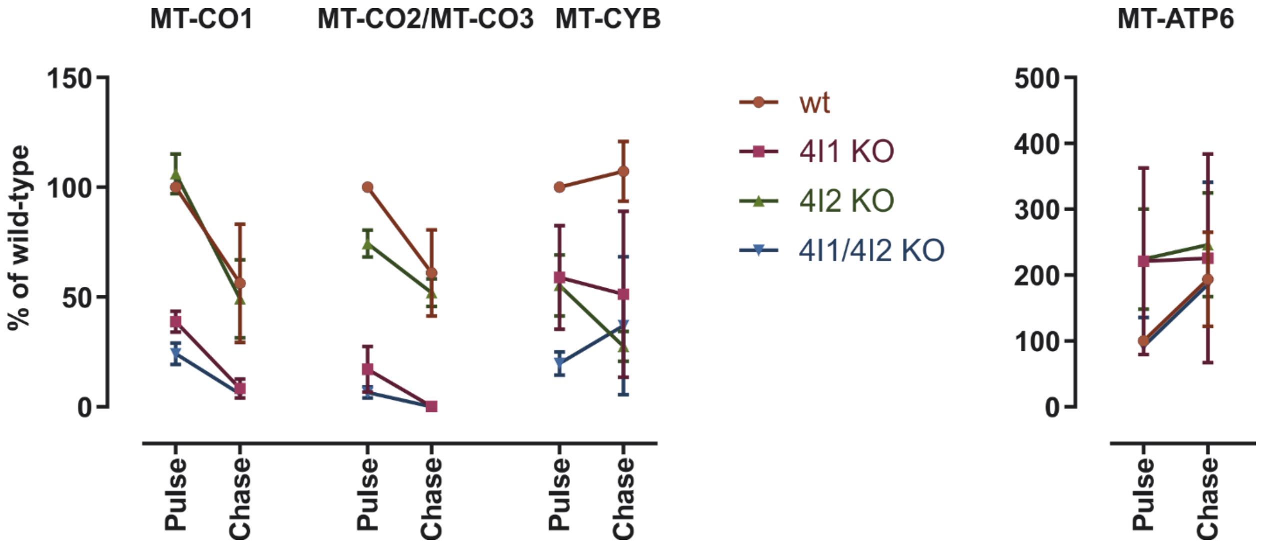

Supplementary table 1. List of antibodies used for Western blot detection.

Protein specifity	Catalogue number	Provider	Dilution	Type
**MT-CO1**	Ab14705	Abcam, UK	1:1000	mo/M
**MT-CO2**	Ab110258	Abcam, UK	1:1000	mo/M
**MT-CO3**	Ab110259	Abcam, UK	1:250	mo/M
**COX4***	11463-1-AP	Proteintech Group, USA	1:1000	rb/P
**COX4I1**	Ab14744	Abcam, UK	1:1000	mo/M
**COX4I2**	H00084701-M01	Abnova, Taiwan	1:1000	mo/M
**COX5A**	Ab110262	Abcam, UK	1:1000	mo/M
**COX6C**	Ab110267	Abcam, UK	1:500	mo/M
**NDUFA9**	Ab14713	Abcam, UK	1:1000	mo/M
**SDHA**	Ab14715	Abcam, UK	1:10000	mo/M
**UQCRC2**	Ab14745	Abcam, UK	1:1000	mo/M
**ATP5F1B**	Ab14730	Abcam, UK	1:2000	mo/M
**ATP5MC1**	Ab18	Abcam, UK	1:1000	rb/P
**actin**	MAB1501	Merck Millipore, USA	1:60000	mo/M
**CS**	Ab129095	Abcam, UK	1:1000	rb/M

COX4* - this antibody is marketed as anti-COX4I2, but in human samples crossreacts with both COX4I1 and COX4I2 isoforms, likely due to large sequence similarity of the two isoforms

Secondary antibodies	Provider	Detection wavelength	Dilution in TBST
**Alexa Fluor 680: goat anti-mouse, donkey anti-goat, goat anti-rabbit IgG**	Thermo Fisher Scientific, USA	700 nm	1:3 000
**IRDye 800: goat anti-rabbit, anti-mouse IgG**	Rockland, USA	800 nm	1:3 000
